# Local impact of temperature and precipitation on West Nile virus infection in *Culex *species mosquitoes in northeast Illinois, USA

**DOI:** 10.1186/1756-3305-3-19

**Published:** 2010-03-19

**Authors:** Marilyn O Ruiz, Luis F Chaves, Gabriel L Hamer, Ting Sun, William M Brown, Edward D Walker, Linn Haramis, Tony L Goldberg, Uriel D Kitron

**Affiliations:** 1 Department of Pathobiology, University of Illinois, Urbana, Illinois, USA; 2 Department of Environmental Studies, Emory University, Atlanta, Georgia, USA; 3 Department of Pathobiological Sciences, University of Wisconsin, Madison, Wisconsin, USA; 4 Department of Microbiology and Molecular Genetics, Michigan State University, Lansing, MI. USA; 5 Vector Control Program, Illinois Department of Public Health, Springfield, Illinois, USA; 6 Fogarty International Center, National Institutes of Health, Bethesda, MD 20892, USA

## Abstract

**Background:**

Models of the effects of environmental factors on West Nile virus disease risk have yielded conflicting outcomes. The role of precipitation has been especially difficult to discern from existing studies, due in part to habitat and behavior characteristics of specific vector species and because of differences in the temporal and spatial scales of the published studies. We used spatial and statistical modeling techniques to analyze and forecast fine scale spatial (2000 m grid) and temporal (weekly) patterns of West Nile virus mosquito infection relative to changing weather conditions in the urban landscape of the greater Chicago, Illinois, region for the years from 2004 to 2008.

**Results:**

Increased air temperature was the strongest temporal predictor of increased infection in *Culex pipiens *and *Culex restuans *mosquitoes, with cumulative high temperature differences being a key factor distinguishing years with higher mosquito infection and higher human illness rates from those with lower rates. Drier conditions in the spring followed by wetter conditions just prior to an increase in infection were factors in some but not all years. Overall, 80% of the weekly variation in mosquito infection was explained by prior weather conditions. Spatially, lower precipitation was the most important variable predicting stronger mosquito infection; precipitation and temperature alone could explain the pattern of spatial variability better than could other environmental variables (79% explained in the best model). Variables related to impervious surfaces and elevation differences were of modest importance in the spatial model.

**Conclusion:**

Finely grained temporal and spatial patterns of precipitation and air temperature have a consistent and significant impact on the timing and location of increased mosquito infection in the northeastern Illinois study area. The use of local weather data at multiple monitoring locations and the integration of mosquito infection data from numerous sources across several years are important to the strength of the models presented. The other spatial environmental factors that tended to be important, including impervious surfaces and elevation measures, would mediate the effect of rainfall on soils and in urban catch basins. Changes in weather patterns with global climate change make it especially important to improve our ability to predict how inter-related local weather and environmental factors affect vectors and vector-borne disease risk.

Local impact of temperature and precipitation on West Nile virus infection in *Culex *species mosquitoes in northeast Illinois, USA.

## Background

West Nile virus (WNv), first reported in the United States in 1999, infects many species of birds as well as humans, equids, and other mammals. Though new to North America, the virus had circulated in Africa, Europe and the Middle East for some time prior to 1999; and other outbreaks, including those in southern Russia, Romania and Israel were indications of a change in the range of the virus [1-4]. Mosquitoes transmit the virus between hosts, with *Culex *species most often implicated as the primary amplification vector [5] and bridge vector [6,7]. Many competent avian hosts have been identified both in the lab and field [8], and recent work in parts of North America have focused on the possible important role of American Robins (*Turdus migratorius*) in contributing to virus amplification and maintenance in the sylvatic cycle [9-11]. The continued risk for this sometimes severe and even fatal disease prompted establishment of annual surveillance programs of virus infection in mosquito populations. Now that several years have passed since the introduction of the WNv in North America, longitudinal data from testing of mosquitoes and host species (including records of human and equine illness) reported through systematic surveillance are available for development of models of the risk of infection. These records can be used to examine differences in infection between and within years and among locations to better understand the risk of transmission of the virus and to predict the possibility of place and time-specific outbreaks.

Though many published reports characterize associations between climatic and landscape factors and WNv occurrence, broad patterns have remained elusive as inconsistent results make generalization difficult. A review of 15 publications identified common landscape variables used to predict risk of WNv transmission, including distance to riparian corridor, vegetation measures, slope, elevation, human population numbers, housing and road density, type of urban land use, race, income, housing age, and host community structure [12-26]. Each predictor variable had a significant relationship with the WNv response variable in at least one study, but the directions of the effects were inconsistent among studies. From our review, we consider these inconsistencies to result largely from differences in climatic factors and mosquito vectors among geographic locations, heterogeneous temporal and spatial resolution of the analyses, and the different response variables used to measure risk.

In studies focused on spatial or temporal risk of illness from WNv, the response variable typically includes data from human disease cases [13,14,16,20,27-29], vector abundance or infection [15,17,30-34], evidence of infection in non-human hosts [21-24,26], or a combination of vector and host data [1,12,25,35-37]. When human case data are the response variable, inconsistencies between WNv risk and environmental factors have been attributed to: exposure occurring away from the home residence; time outdoors; use of insect repellent; socioeconomic differences, and even variable case definition [38-41]. An analysis in Indianapolis, IN, with similar climatic features and spatial scale as the current study, identified a relationship with both low precipitation and higher temperatures and increased numbers of human WNV cases [36].

For analysis of the transmission of mosquito-borne arboviruses, vector abundance and infection data are key elements, and the two are not always proportional in space or time [42,43]. For this reason, the fine scale patterns relevant to production and dispersal of adult female mosquitoes and the association between WNv infection in mosquitoes and weather in the context of other landscape features are in need of further investigation. When analyses include different vectors, results will often be inconsistent [16,29]. For example, the population size of *Culex pipiens*., the primary enzootic and epidemic vector in the eastern U.S. north of 36 degrees latitude, is often impacted negatively by large rain events due to the flushing of catch basins, a primary urban larval habitat, and the reduction of organic content in all ovipositing sites [44,45]. By contrast, the vector *Culex tarsalis *generally responds positively to heavy precipitation, which provides the typical larval habitat in rural areas in the western part of the U.S [28,33]. In semi-permanent wetlands, drought conditions can increase abundance of some vector populations as they result in more larval breeding sites with fewer competitors and mosquito predators [46]. Early season drought with subsequent wetting and low water table depth preceded amplification episodes for both WNv and St. Louis encephalitis virus in peninsular Florida, where *Culex nigripalpus *functions as the main vector [47-49].

Increased temperature is known to increase growth rates of vector populations [35], decrease the length of the gonotrophic cycle (interval between blood meals), shorten the extrinsic incubation period of the virus in the vector and increase the rate of virus evolution [50-54]. Kunkel et al. [55] showed a correlation between the number of days when daily maximum temperature exceeded a threshold (degree days), timing of a seasonal shift to a higher proportion of *Culex pipiens *among all *Culex *species, and the onset of the amplification phase of WNv transmission seasonally in Illinois. Other studies considered less proximate weather conditions, such as increased rainfall in the preceding year [56]. Warmer winter temperatures and warmer March and April may lead to larger summer mosquito populations [32]. Temperature has also been linked to the rate of evolution of the virus and warmer temperatures facilitated the displacement of the WNv NY99 genotype by the WN02 genotype [53]. Bertolotti et al. [57] discovered high genetic variation of WNv at fine temporal and spatial scales, with variation in local temperature offered as one explanation for it. At the same time, in one study, very high temperatures (above 30°C) reduced larval *Culex tarsalis *survival [58].

Finally, the choice of geographic scale and units can impact profoundly the outcome of an analysis. Coarse geographic scales such as the county level can obscure fine spatial patterns in a heterogeneous landscape [38]. The census tract provides a somewhat finer scale geographic unit, but while logistically useful, is not a biological meaningful unit. In fact, any use of a spatial unit introduces the modifiable area unit problem [59,60], where spatial units of analysis become arbitrary or possibly even introduce systematic bias. In addition to the importance of fine spatial scales, coarse temporal scales could obscure some temporal patterns [31,32].

Our analysis focuses on greater Chicago, Illinois, where WNv infection in mosquitoes, horses and birds was first noted in 2001, and where human illness has been reported every year from 2002 to 2009 [[13], http://www.idph.state.il.us/envhealth/wnv.htm]. We consider temporal and spatial patterns of infections in mosquitoes for the years 2004 to 2008, years for which comprehensive mosquito testing data were available in Illinois. We have focused, in particular, on the meteorological conditions that precede or are concurrent with amplification, when a sharp increase is seen in the infection rate in mosquito populations. Our three research questions are: 1) Inter-annually: what are the conditions associated with higher mosquito infection in some years compared to others? 2) Intra-annually: what temporal characteristics of rainfall and temperature precede changes in mosquito infection and with what temporal lag? 3) Spatially: can the patterns of rainfall and temperature help explain the differences in mosquito infection across space? We used surveillance data from the Illinois Department of Public Health (IDPH) and publicly available meteorological readings, and consider the heterogeneity of urban land cover through an analysis of digital spatial data to identify and forecast favorable conditions for WNv amplification in the greater Chicago area.

## Methods

### Mosquito database

**Figure 1:**
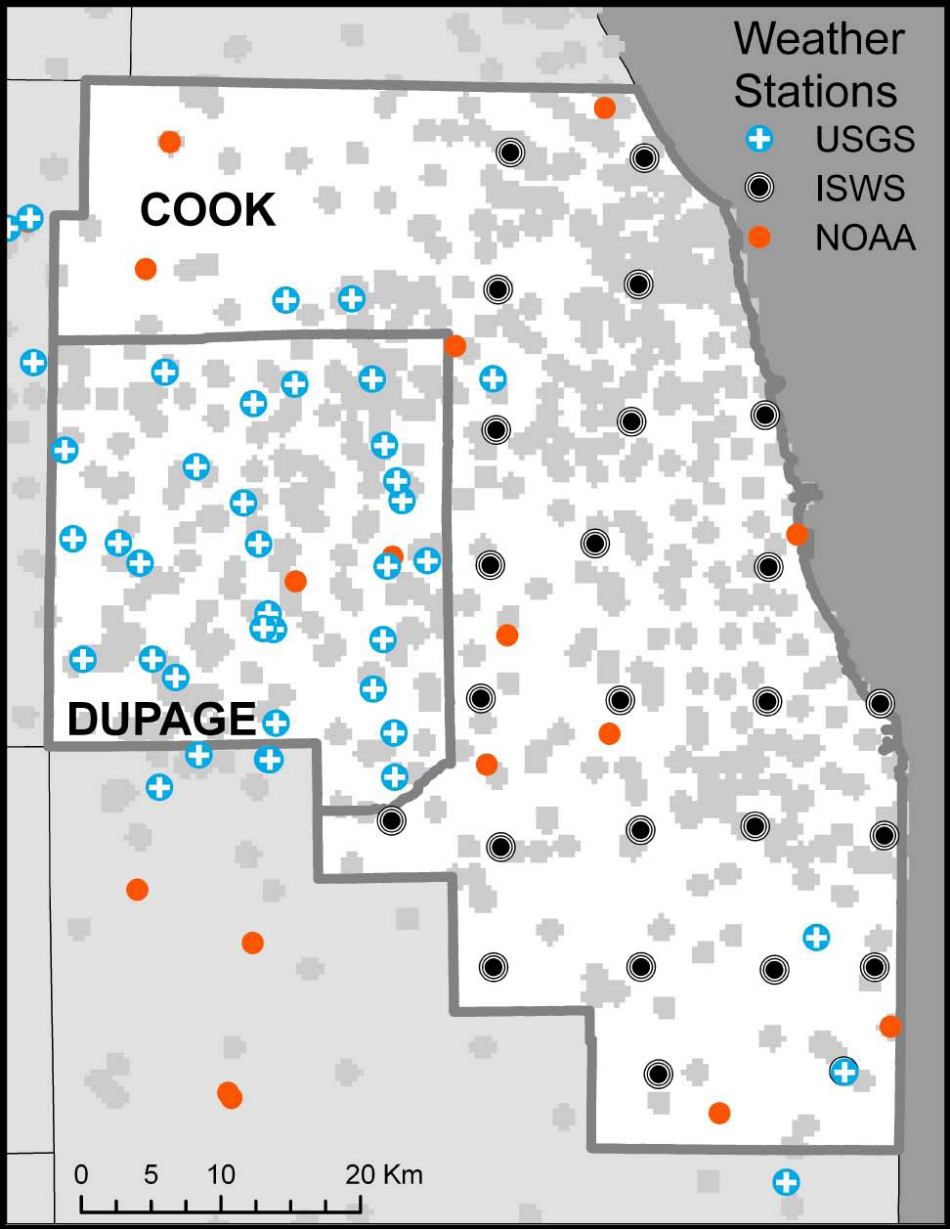
**Study area in the greater Chicago, Illinois**. General area of mosquito trap locations for 2004 to 2007 (grey shaded areas) and locations of all weather stations with data for the years under analysis are included. Cook county and DuPage county are the area of analysis, while data from the five surrounding counties provided additional support for the spatial interpolation of variables. Mosquito trap locations are shown as gray areas to be illustrative of the general pattern of the traps, rather than to show specific locations, which varied somewhat from year to year. Of the 1263 hexagons (not shown) used for the spatial analysis, 409 (32%) had a trap location in them in at least one of the years.

To measure mosquito infection, we used data from the Chicago metropolitan area (Cook and DuPage counties, Illinois, USA) extracted from a statewide database of mosquito pool test results maintained since 2004 by the Illinois Department of Public Health. Tests for WNv from all *Culex *species mosquitoes for the years 2004 to 2007 were included in our empirical analysis to develop the temporal and spatial models. The same data from 2008 were used to test the ability of the temporal model to predict weekly infection patterns. The data reported for the two counties were organized by pools of mosquitoes (modal batch size of 50) and included 7,788; 7,067; 9,321; 11,842 and 9,024 pools tested for the years 2004, 2005, 2006, 2007 and 2008 respectively. Tests used to measure infection included VECTEST, PCR and RAMP test methods. For the purposes of the analysis presented here, only one test result per pool was used as an indication of infection, with PCR results taking precedence over the other two methods. Pool locations were geocoded to a street address, using ESRI StreetMapUSA and ESRI geocoding (ESRI, Redlands, CA). Those addresses that did not geocode automatically were located manually using Google Earth or by identifying landmarks found in the database. The exact number of trap locations varied by year and by week. There was an average of 370 unique trap locations in the two-county area during the years in question (Figure 1). The pool test data were averaged by trap location and by week, where the week coincided with those used by the CDC for reportable diseases http://www.cdc.gov/mmwr. The mosquito infection rate for each week and for each trap location was calculated using the CDC Excel add-in for calculation of pooled infection rates [61], using the Minimum Infection Rate (MIR) method. We subsequently capped the raw MIR values at a value of 76.92 (the 95^th ^percentile for the weekly non-zero MIR values) prior to further spatial processing to reduce the effect of very high outliers possible with MIR.

### Weather database

Weather data from archived sources from the two counties and five surrounding counties were retrieved from three sources (Figure 1). The NOAA NESDIS National Climatic Data Center http://cdo.ncdc.noaa.gov/CDO/cdo included from 21-32 stations, depending on the year, and provided both temperature and precipitation. From the U.S. Geological Survey, we retrieved data from 18 to 41 stations per year, all reporting precipitation http://pubs.usgs.gov/wdr/2005/wdr-il-05/start.htm. Additional precipitation data were obtained from a network of 25 stations maintained by the Illinois State Water Survey http://www.isws.illinois.edu/atmos/ccprecipnet.

Temperature data were further processed to create a variable of degree week (DW), fashioned after the concept of the more common degree day. The DW was calculated as(1)

where Tmean is the average temperature in a week and Tbase = 22 deg C. The Tbase (threshold temperature) represents a weekly mean temperature that might affect growth or activity of an organism and was chosen empirically as the value that was most correlated with the mosquito infection rate [55,62]. The selection of this value is explained more fully in the Results section (below). In order to estimate the MIR when it was declining, we also used a Cooling Degree Week (DWC), with a Tbase of 22 deg C. The DWC was calculated as(2)

In addition to the single weeks' precipitation measures, the weekly precipitation was smoothed using a 3-week and a 5-week moving average. These three different weekly measures of precipitation were included to provide several levels of smoothing for this variable.

### Other environmental factors and geographic units

For spatial analysis, we created a 2000 m hexagon grid encompassing the two county study area and summarized all data for those units (N = 1,263). The hexagon size was chosen to equal about the same number of units as census tracts in the same area, but in contrast to tracts are of uniform size, providing a more neutral landscape unit. For mosquito infection, precipitation and temperature, we used a local moving average method of geographic interpolation [63,64] with inverse distance weighting (IDW) from the six closest points to create weekly GIS raster grid (100 m grid size) maps for each week and for each variable. The "Z" values (also known as the support) for the IDW interpolation were based on the calculation of the MIR at the trap locations or the weather variables at the weather stations for which data were available for that week. For this step, we included data from five counties bordering Cook and DuPage counties to better estimate values at the edge of the study area. In this way, we estimated values for each raster grid in the study region. The raster grid-based data were then summarized for the 2000 m hexagons with ArcGIS using the average of all raster grid cells within each single hexagonal geographic unit.

We developed a spatial database of environmental factors included previously in an analysis of human illness risk in this area [13,65] to assess the spatial differences in MIR relative to both weather-related and other environmental factors. While the MIR and the weather data were summarized by week, other environmental factors did not have a temporal component and their values were static. To account for the social environment, we included the number of people in residence in each hexagon, the percentage of the population defined racially as "Black" in the U.S. census, and the percentage of housing from different decades [66]. We suggest that both mosquito control and mosquito production vary by neighborhood due both to engineering and housing development differences related to age of housing and socio-cultural differences that affect vegetation choices and landscape patterns. Terrain characteristics were measured by the minimum, maximum, range, mean and standard deviation of the elevation from a digital elevation model http://seamless.usgs.gov. These terrain variables provided several ways to measure the degree to which water would flow or remain in an area. For land cover, we measured the mean and standard deviation of imperviousness (landcover.usgs.gov), and the percentage and standard deviation of percentage vegetation (per hexagon) from a digital land cover map http://www.agr.state.il.us/gis/landcover99-00.html. These final variables accounted for the amount of vegetation and the amount of surfaces where soil was covered by pavement. We expected those to be related to local mosquito and bird habitat.

### Statistical Methods

We performed correlations and linear regression as well as classification and regression tree analysis using the R statistical package [67]. For the first and second research questions, we considered the difference of the weekly MIR from the average MIR at that week across years, as well as the difference in temperature and rainfall from their respective long term means. In this way, we accounted for the patterns that were outside the dominant annual cycle of solar radiation and the seasonality component, which remains unchanged in every year, and focused on the components deviating from this fixed annual cycle. To calculate these differences, the expected, or normal, value of mosquito infection for a given week in the Chicago area was needed. It is not easily known since WNv has been in the area for a relatively short time and there can be instability of MIR results [68]. For MIR, we defined the "normal weekly value" as the average for each week in the combined years 2004 to 2007 as measured from the hexagon level dataset. For temperature and precipitation, the 30-year normal weather values are an accepted standard, but we also wanted to consider the specific differences and possible warmer trends of the more recent years in question. Thus, we defined normal weekly weather values both as the average weather values between the years 2004 and 2007, and the 30-year normal values. We then compared the two results. The 30-year normal values were detrended by accounting for the linear change over the years.

Time-lagged correlations were used to explore the association between mosquito infection and weekly temperature and rainfall at a range of temporal lags. Linear regression models were then used to estimate the empirical models that best fit the MIR with weather variables at key temporal lags selected based on the exploratory correlation analysis. Weekly weather variables were considered starting in week 1, while for MIR, we focused on the weeks from 18 to 35 (from about the end of April to the end of August), during the period of increasingly stronger activity and up to the peak values of MIR. We developed linear models with and without an autoregressive term for MIR, so that if MIR was already known, then the prior week's MIR could be included in the estimate for the following weeks. We developed several linear models and used them to reconstruct the weekly pattern of MIR for the years 2004 to 2007. These models were used then to predict the weekly pattern for 2008, thus allowing a test of the model against a year not included in the model development.

For the third research question, we used regression tree analysis (RT) and Random Forests (RF) with weekly temperature and precipitation and the other environmental factors as measured in the hexagon grid. With RT analysis, a set of rules is developed from the independent (or predictor) variables that can best recreate the observed pattern in the response variable [69]. The response variable in this analysis was the average MIR during the period of peak infection, weeks 32 to 34. In this technique the variability of the response variable is partitioned along binary nodes of the predictive covariates that will lead to an average value of the response variable. Nodes of covariates can be nested, with the most basal explaining most of the variability in the response variable. This technique has the advantage over traditional generalized linear models of capturing non-linear relationships between the covariates and response and has no assumptions about spatial or temporal autocorrelations. It has been used to analyze data in other mosquito-borne disease systems [70]. An additional advantage of this technique is that it doesn't rely on the assumptions that are required for parametric statistics and the analysis is not restricted by linearity in predictor or response variables or by multicollinearity in predictor variables. Trees were selected using the "cost-complexity" algorithm, where auxiliary nodes are cut if no significant loss in the mean square error of the predictions is detected. This technique helps to avoid over-parameterization.

RF is a tool for prediction based on several regression trees. Basically, a new response is constructed using the fit of a decision tree using sampling with replacement. A new decision tree is built, and predictions are based on the aggregate outcome of the trees forming the random forest [71]. Here we used fully cross-validated regression trees, which involves leaving out one of the observations when fitting a tree or a forest and measuring the error (difference) between the observed and the predicted value for the observation not used for the fit. Random forests with 1000 trees were used to predict average MIR during the period of high infection, in weeks 32 to 34, for the years 2004, 2005, 2006, and 2007. We analyzed all years together and separately, and used as covariates: 1) all weekly weather and other environmental factors, 2) only weather variables, and 3) only other environmental factors.

## Results and Discussion

Research Question 1) Inter-annually: what are the conditions associated with higher mosquito infection in some years compared to others?

### Descriptive overview of mosquito infection and weather

The year 2002, when the first cases of human illness were reported in the two counties, had the largest number of total cases for all years, at 686 (Table 1). Some cases have been reported each year since but human illness and mosquito infection were notably high in 2005 and 2006 compared to 2004, 2007 and 2008. The years with larger numbers of cases (2002, 2005 and 2006) also had warmer than average weather during the period prior to week 35, but only 2002 and 2005 had less than average precipitation. The accumulated Degree Week (DW) value at week 35 was highest in 2002 and 2005, at approximately 30, or five times higher than the lowest DW of 6, in 2004. The amount of rain was especially low in 2005 (2/3 of normal).

**Table 1:** West Nile virus infection and weather for Cook and DuPage county from 2002 to 2008.

YEAR	Average mosquito infection (MIR)	Percent *Culex *mosquito pools positive	Number human cases illness	Annual precipitation in cm (difference from average)	Degree weeks at week 35 base = 22 C
2002			686	85.7 (-9.4)	30.0

2003			23	88.6 (-6.5)	15.2

2004	2.6	12%	28	88.2 (-6.9)	6.1

2005	4.5	19%	182	65.2 (-29.9)	31.2

2006	4.0	17%	129	117.8 (22.7)	25.7

2007	2.0	7.6%	53	100.1 (5.0)	17.9

2008	1.3	6.5%	9	126.6 (31.5)	12.5

Average annual precipitation for the weather stations in the area was 95.1 cm using 30-year normal measures.

**Figure 2:**
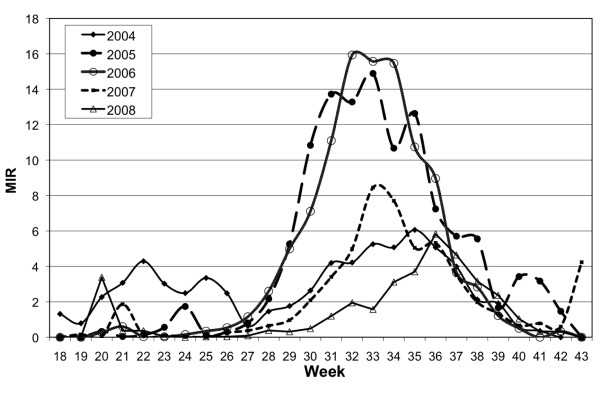
**Mosquito infection by week, 2004 to 2008, in Cook and DuPage counties in Illinois**. The values reflect the minimum infection rate calculations averaged from the 2000 m hexagons used in the regression models.

In the weekly patterns of MIR, both 2005 and 2006 have a unimodal pattern with peak MIR at week 33, and the maximum weekly MIR for those years was above 14 per 1000 mosquitoes (Figure 2). In the year 2004, the mosquito infection rate was low, and the pattern was bimodal, with a relatively strong early peak. The years 2007 and 2008 both had low virus activity, with 2007 being distinguished by a late season increase in mosquito infection at week 42 and 2008 having a peak about one week later than other years.

Research Question 2) Intra-annually: what temporal characteristics of rainfall and temperature precede changes in mosquito infection and with what temporal lag?

### Temporal model - exploratory phase

**Figure 3:**
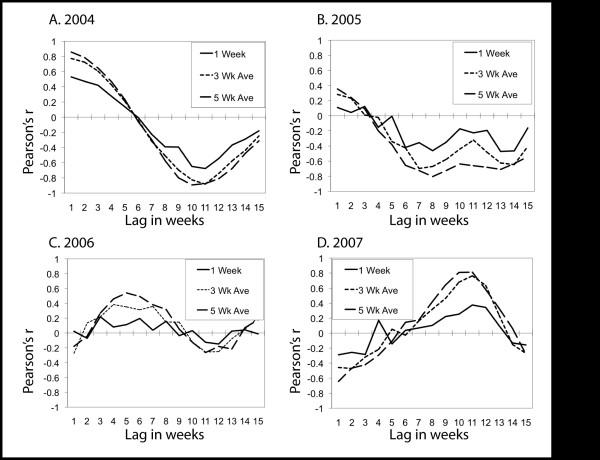
**Correlation between weekly MIR and precipitation**. Weekly values are averaged on 1, 3 and 5 weeks at different lags during each year from 2004 to 2007 in Cook and DuPage counties, Illinois. There are 18 weeks with 15 possible temporal lags for each year.

When we compared the variation in MIR and precipitation (using the 4-year average to calculate differences) at all temporal lags for each year, we observed a strong negative correlation between MIR and precipitation about 10 to 12 weeks earlier in the years 2004, 2005 and 2006 (Figure 3; Additional File 1: Exploratory, Figure A). This did not hold true in 2007, however, where there was a strong *positive *correlation between precipitation and MIR about 10 weeks prior to the MIR values. We also noted evidence in 2004 and 2005 of positive correlation at 1 to 3 week lags between precipitation and MIR. When the four years are combined, the general trend is for a statistically significant negative correlation at the 11-week lag using the 5-week moving average measurement (r = -0.4, p < 0.01, N = 72). Correlations measured between MIR and weather using weather variables measured as the difference from the 30-year normal for these same conditions were virtually the same (r = -0.39, p < 0.01, N = 72) and the 4-year average measures were used henceforth. Overall, these results suggest that drought followed by wetting may be associated with higher MIR in some years. The difference among years is notable, indicating that precipitation measures alone are not sufficient to predict the timing of amplification of the virus.

**Figure 4:**
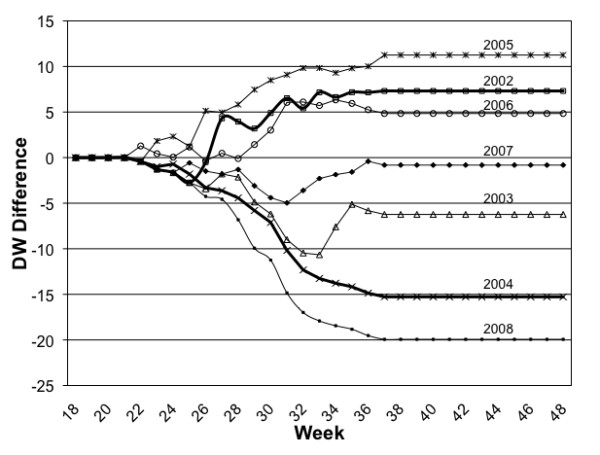
**Weekly Differences in Degree Weeks**. Cumulative Degree Week values defined at Tbase = 22°C and portrayed as the difference from average for the years from 2002 to 2008.

We then examined relationships between temperature and MIR and determined the optimal Tbase value for the Degree Week (DW) variable where this correlation was maximized. With all four years combined, the correlation coefficient between the accumulated DW starting from week 1 and the MIR from weeks 18 to 35 varied between -0.5 to 0.8 at lags from 1 to 11 weeks with DW defined at all possible Tbases. The correlation maximized at 0.80 at the point where Tbase = 22°C and the accumulated DWs lead the MIR by 1 week (r = 0.80) (Additional file 1: Exploratory, Figure B). This guided the decision to use the base value of 22°C in the calculation of the Degree Week variable and to select the temporal lag of 1 week for use in the development of the linear model. The deviation of the accumulated DWs from its detrended 30-year average, and its correlation with the MIR was not very different from the 4-year average, and again maximized at approximately Tbase = 22°C when the accumulated DW leads by 1 week (r = 0.77). Finally, we observed that when years are compared by their DW difference from average values (based on the 4-year average) across weeks, the years with higher differences in DW are also those when both MIR and human illness were higher (Figure 4 and Table 1). The differences in DW between higher and lower MIR years is most clear in weeks 29 to 33, when amplification is most likely to occur.

### Temporal model -regression model development phase

Using the variables indicated from this exploratory analysis, we developed linear regression models to determine the best-fit model for all of the years. We used these to reconstruct the weekly changes in MIR for the years 2004 to 2007 and to simulate weekly changes for the year 2008, which was not included in the model development. We also simulated MIR for the years 2002 and 2003, years for which comparable mosquito data were not available, to learn what patterns would be estimated for a year with high human illness (2002) and a year with few cases (2003). Note that both the independent and dependent variables in the regression models are the differences from the corresponding averages between 2004 and 2007 but for simulated values, the averages were added back in.

The candidate variables for this set of models, based on the exploratory phase, were DWs accumulated from the beginning of the year up to one week prior to the MIR measure (*DW*), the 5-week-average precipitation 11 weeks prior (*prcp5wk_lag11*) and the 3-week average precipitation 3 weeks prior (*prcp3wk_lag3*). Further, considering that in other studies the human WNv incidence was associated with annual precipitation from the preceding year, we included that variable as one of candidate variables (*prcp_annual*). Finally, we found that the MIR itself is a first order auto-regressive (AR) process, so to simulate the MIR for any specific week, the MIR measured in the previous week was also included as one of the possible explanatory factors. After developing the best-fit models, we also created models that were somewhat less strong, but would allow for prediction earlier than the best-fit models. For these, we considered models both with and without the AR term.

Two initial linear regression models were thus developed (Model 1 and Model 2 in Table 2). For these two models, the variables were selected by AIC via backward selection from the candidate variables. Model 1 included an AR term for MIR, while Model 2 did not. The R^2 ^of model 1 is 0.8, and the coefficients related to the *DW *and AR terms are significant at α = 0.05 and the coefficient for *prcp_annual *was significant at α = 0.1. Model 2 was developed by the same approach, but without the AR term. The R^2 ^of this model was 0.70, and coefficients related to three explanatory factors *- DW, prcp_annual *and *prcp3wk_lag3 *are significant at α = 0.05.

**Table 2:** Coefficients and variables from temporal regression models.

	Model1	Model2	Model3	Model4	ModelC
**Auto-regression**					
1^st ^order	0.64*				0.31*
2^nd ^order			0.61*		
**Precipitation**					
prcp3wk (3 wk lag)		0.35*			
prcp3wk (4 wk lag)				0.43*	
prcp5wk (11 wk lag)					
prcp_annual (prior year)	-0.78**	-1.57*	-1.30*	-1.89*	
**Temperature**					
DW (1 wk lag)	0.16*	0.42*			
DW (4 wk lag)			0.21*	0.59*	
DWC (1 wk lag)					-0.08*
**R**^**2**^	0.80	0.70	0.65	0.58	0.42

Model 1 and Model 2 measured the effect of weather on mosquito WNv Minimum Infection Rate (MIR) and the best statistical models first with and then without an autoregressive term (AR) for MIR. Models 3 and 4 are less robust statistically but estimate MIR using weather conditions at earlier points in time to provide forecasting. The Model C models the cooling period, after amplification and includes only one option of variables (Additional File 2: Temporal, Part C includes the full equation for each model).

*p-value < 0.05

** p-value < 0.1

For Models 1 and 2, we found that the precipitation variable *prcp5wk_lag11 *was not selected as an explanatory factor, though it had been found to be strongly negatively correlated with the MIR based on the correlations. This can be explained by its co-linearity with the DW variable (r = -0.7). Specifically, years with a dry spring season also tended to be hot during that period (Additional File 2: Temporal, Figure A). Thus, adding *prcp5wk_lag11 *did not significantly improve the model.

As noted above, the MIR in our study area peaked around week 34, after which it decreased rapidly until reaching zero at around week 43. Although the year-to-year contrasts during this declining period were not as striking as those in the growth period, we still found some inter-annual variation. For example, MIR decreased at a lower rate in the fall of 2006 and 2007 than in other years. We found these differences can be explained by the higher than normal temperature during that period in those years. Thus we constructed a linear regression model (Model C in Table 2) for the MIR in its declining phase using the cooling DW (*DWC*) and the AR term.

**Figure 5:**
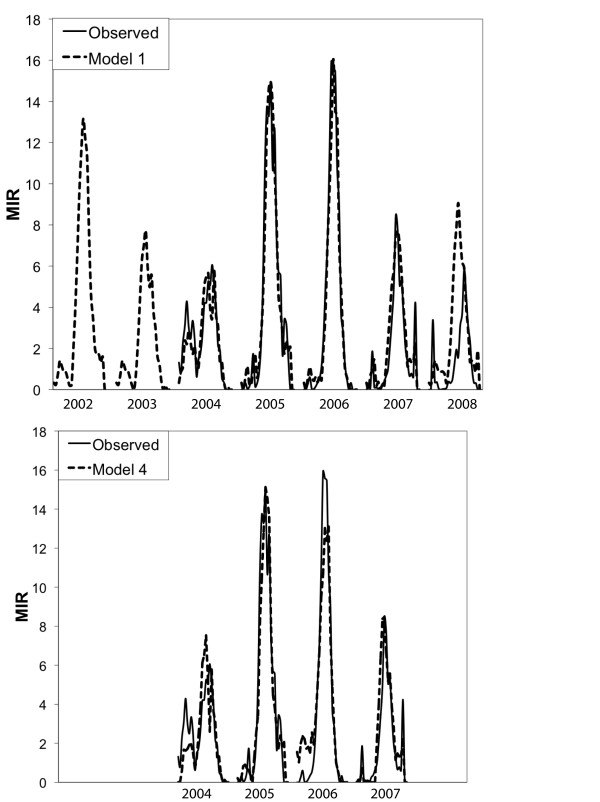
**Estimated and observed mosquito MIR from 2002 to 2008**. Lines shown are from observations (solid line) and simulations (dashed line) for Model 1 and Model 4. Only MIR between 2004 and 2007 were simulated by model 4, and MIR in 2008 was only simulated by model 1.

We then reconstructed MIR by adding back the average MIR calculated from Model 1 and 2 and the cooling model to its 4-year mean (Figure 5A). The model of simulated MIR agreed with the observations very well. We also reconstructed MIR for 2008 - a year not used to estimate the model. The simulation for 2008 predicted an earlier and higher peak than in the observed data, but was, correctly, more like the other low-infection years than a high-infection year.

### Forecasting the MIR temporally

An early warning system for mosquito infection from WNv will be most useful when it is possible to estimate mosquito infection as far in advance as possible. Models 3 and 4 were developed for this purpose. Here, earlier meteorological information was used to forecast the MIR to facilitate decisions on corresponding preventive measures (Table 2). While the highest correlation between the accumulated DW and MIR was at a lag of 1 week, the DW value cumulated to 4 weeks prior was also strongly correlated, at around 0.7. For Model 3, we included the accumulated DW value leading by 4 weeks, the AR term with a lag of 2 weeks and the precipitation from the prior year as forecasting factors for MIR. In the event that only meteorological variables were available and no prior MIR was included, then the 3-week average precipitation at a 4-week lag, the accumulated DW at a 4- week lag and the prior year precipitation provides a less strong but adequate forecasting model as seen in Model 4 (Table 2 & Figure 5B).

Sensitivity analysis of the four models based on both leave-one-out cross validation and an approach using 5-fold cross validation both indicated that the predicted R^2 ^was nearly identical to the model R^2^. Model residuals were found to be nearly normal in Model 1 and without temporal autocorrelation. Model 1 included a 1^st ^order auto-regressive term on MIR (Additional File 2: Temporal, Figure B), but the pattern of residuals for other models without that auto-regressive term did have evidence of the need for this term. The MIR for a prior time period may be necessary to build robust weekly models. The differences seen in the model diagnostics between years highlights the difficulties in finding general trends across only a few years, in a region where weekly weather patterns can diverge considerably from year to year.

Research Question 3) Spatially: can the patterns of rainfall and temperature help explain the differences in mosquito infection across space

### Spatial patterns of MIR

**Figure 6:**
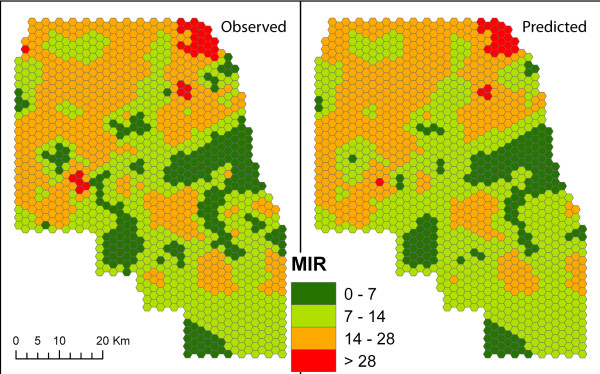
**Mosquito infection measured from observed and spatial model estimated data**. The mosquito infection was measured for weeks 32 to 34, 2005, and the infection rate predicted by the random forest model using all variables (right) for the same time period.

We found that random forests (RF) (Table 3 & Figure 6) in general outperformed regression trees (RT) (Table 4). This difference is especially clear when looking at the trees and random forests built for all years studied, where the R^2 ^of the forests that included climatic covariates is above 70%, while the best RT has an R^2 ^slightly below 50%. For both RTs and RFs, models including climatic covariates outperformed those based only on landscape and demographic covariates. For the RF models, those including only climatic covariates outperformed those that also included the landscape and demographic predictors. There was also great variability in the explanatory power of the models across years. For 2004, the covariates had the highest explanatory power (above 75%) (Additional File 3: Spatial), followed by 2006, 2005 and 2007 (all above 60%). The most important variables for models including all variables, were the weather variables. These were selected based on their ability to decrease the R^2 ^if not considered when fitting the models. For the landscape and demographic models, elevation, impervious surfaces and percent black were the most important. The direction of these relationships is not always clear from our analysis but suggest that the role played by these is due to neighborhood characteristics that affect mosquito production or transmission dynamics. For the full models (i.e., those with climatic and other covariates) and the models with weather alone, precipitation and temperature in early weeks of the mosquito season (weeks 16-20) and close to the peak of transmission (weeks 30-33) were the most important to explain the spatial MIR patterns.

**Table 3:** Random forest spatial model results.

Variables Tested and Model Type	Year	R^2^	Most important variables in model
All variables Random Forest	All		Precip W33, W19, W28
			Temp W27
		76.89	Pct Black
	
	2004	81.77	Precip W23, W29, W33
	
	2005		Precip W28, W21
		64.48	Temp W33
	
	2006	74.76	Precip W29, W18, W19, W33
	
	2007		Precip W15, W28
		62.88	Temp W17

Weather variables only Random Forest	All		Precip W33, W19
		78.77	Temp W27
	
	2004	82.99	Precip W33, W23, W29
	
	2005	70.55	Precip W32, W28, W19
	
	2006	77.03	Precip W33, W29, W19
	
	2007		Precip W15, W21
		67.01	Temp W17

Non-weather variables only. Random Forest	All	13.27	Pct Black, Human population, Elevation range
	
	2004	47.34	Pct Black, Maximum elevation, Minimum elevation
	
	2005	34.47	% pre-40's housing, Pct Black, % 50's housing
	
	2006	42.27	Pct Black, maximum elevation, % 90's housing
	
	2007	37.95	Pct Black, maximum elevation, mean elevation

The R^2 ^value indicates the ability of random forests to predict mosquito infection in weeks 32 to 34. Also included are the most important variables from the models listed in order of importance. Results are divided according to which variables were included in the models. See Additional File 3: Spatial, for a regression tree graphic.

**Table 4:** Regression tree spatial model results.

Variables Tested and Model Type	Year	R^2^	Most important variables in model
All variables Regression Tree	All		Precip W17, W19
		48.24	Temp W35
	
	2004		Precip W29, W28
		70.95	Temp W28
	
	2005		Temp W33
		55.57	Precip W24, W26
	
	2006	67.22	Temp W35, W27
			Precip W19
	
	2007		Temp W17, W22
		61.63	Mean elevation

Weather variables only Regression Tree	All		Precip W17, W19
		48.24	Temp W35
	
	2004		Precip W28, W29
		68.67	Temp W28
	
	2005		Temp W33
		56.27	Precip W24, W26
	
	2006		Temp W35, W27
		67.99	Precip W19
	
	2007		Temp W17, W22
		61.16	Precip W15

Non-weather variables only Regression Tree	All	8.28	Impervious surface, % pre-40's housing, % 50's housing
	
	2004	47.41	Minimum elevation, Mean elevation, impervious surface
	
	2005	32.23	Maximum elevation, impervious surface, Human population
	
	2006	48.83	Maximum elevation, impervious surface, Human population
	
	2007	42.03	Maximum elevation, impervious surface, Human population

The R^2 ^value indicates the ability of random forests to predict mosquito infection in weeks 32 to 34. Also included are the most important variables from the models listed in order of importance. Results are divided according to which variables were included in the models.

## Conclusions

Finely grained temporal and spatial patterns of precipitation and air temperature have a significant impact on the timing and location of increased mosquito infection across the Chicago study area. Temperature, in particular, mediates the magnitude and timing of increased MIR within a season and it is strongly indicated as a key factor for explaining much of the observable differences between years. The effect of increased temperature on MIR is especially strong within a week. This is consistent with expectations and would suggest increased mosquito productivity through a shortened time for development of the *Culex *vector and increased rates of oviposition activity [72], which implies increased biting rates for anautogenous populations. Further, more quickly replicating virus spurred by higher temperatures precedes an increase in mosquito infection. The patterns of association between MIR and precipitation for 2004, 2005, and 2006 are similar to the patterns observed in Florida, where periods of dry-downs and wetting events lead to elevated SLE and WNV infections [48,49]. In Illinois, however, 2007 demonstrated a strong departure from this pattern and further emphasizes the more important role of temperature. Additional years of data on mosquito infection and weather need to be assessed to better determine the dominant patterns.

The lag seen in the effect from rainfall and differences in this effect among years suggests that the relationship with rainfall and MIR is more complex and more variable than the effects of temperature. The observed lower rainfall about eleven weeks prior to the highest MIR weeks (32 to 34) indicates that the processes in play may be related to subtle changes in soil moisture, near ground humidity, and an increase in desirable oviposition sites that would result from an increase in organic matter in standing water. While dry years were more likely to result in higher MIR, both higher temperatures and less rainfall in tandem were more highly associated with the highest mosquito infection rates and/or with more human cases. This was seen in particular in the years 2002 (only human illness data available) and 2005 (evidence from human illness and mosquito infection).

WNV infection in mosquitoes was negatively correlated with the previous year's precipitation. This is contrary to the findings of Landesman *et al*. [56] for the eastern part of the United States, and consistent with those in the west. The mechanisms for the patterns between past weather conditions to influence arbovirus transmission are still unclear. Our results support the importance of using off-season or previous year weather data in prediction models, but details on this must be left to a future analysis with a longer time series [32]. We also suggest that it is worthwhile to consider winter temperature conditions, which might lead to a larger number of overwintering adult *Culex *spp. mosquitoes and increase the potential for amplification the following year due to larger initial vector populations.

Compared to the temporal patterns, the spatial analysis indicated the relative importance of rainfall, with temperature playing a more muted role. The results for the 2004 random forests for example (the strongest of the models) show that the three most important variables all measured precipitation. The more consistently seen measures of importance indicated that drier and warmer conditions tended to result in higher MIR, but many exceptions also occurred, emphasizing the complex nature of the spatial patterns. The spatial models for a single year were consistently stronger than those where data from all years were combined. From a public health perspective, this would indicate that the infection risk changes from year to year as weather patterns vary. This is consistent with the outcome of the temporal models, which also exhibited different patterns by year. At the same time, the spatial models for all years were modestly robust. From this, we conclude that in balance, in future efforts to determine factors that influence MIR, it is more important to focus on the dynamic weather variables, possibly supplemented by more dynamic measures of vegetation and surface moisture, rather than the more static landscape metrics.

In terms of the environmental features that were found to influence MIR, it is worth noting that the more important non-weather variables were the proportion of impervious surfaces and elevation variables. Both of these factors would mediate the effect of rainfall on soils and vegetation and in catch basins. For example, low-lying landscape patches with some impervious surfaces could be expected to be more productive for mosquitoes, since they can accumulate water and have resources necessary for mosquitoes to thrive. The importance of the spatial variable that measures the percentage of the population identified as "Black" by the U.S. Census Bureau was also notable, as this was a variable of note in prior analyses in this region, which focused on human case data [13,65]. Though human case data would be influenced by possible biases due to differences in reporting rates in different neighborhoods, mosquito infection rates are a more neutral measure of presence of the virus. While the present analysis did not allow us to fully explore this relationship, the persistence of the statistical association in the Chicago area warrants further investigation.

We further suggest that a finer grained spatial measure of temperature than that used here may also reveal that temperature plays a role that was not detected with the resolution of temperature data available. Especially, given the strong importance of temperature in the temporal models, differences in surface temperatures due to the amount of vegetation relative to the built environment may be more important than what was revealed in the current analysis. This can be addressed by using remote sensing techniques to obtain more complete continuous coverage of temperature than what is possible with data from point-based weather stations.

The ability of the weekly models to simulate MIR based on prior precipitation and temperature and prior MIR is moderately strong and indicates that at least on a weekly basis, the amplification of the WNv in mosquitoes can be forecast for this area when the level of mosquito infection from the prior week is available for the model. However, given the practical and statistical difficulties in using fine-scale time series data, a coarser temporal scale may be more realistic from a public health standpoint.

Local forecasts available to public health personnel would need to have parameters specific to the place of interest. By way of comparison to our own results, increased temperatures in a *Culex pipiens-*driven system in Israel resulted in a significant two-week lag for mosquito abundance and about a five week lag for human cases of WNV [35]. Maximum urban and grass temperature were strong predictors of human cases of WNV in the Chicago, Illinois region and low precipitation and warm temperature were associated with WNV cases in Indianapolis, Indiana [36]. Each of these examples further emphasizes the need for models that are place-specific. The framework for developing early warning systems needs to be standardized but geographic variability makes it necessary to customize the system for each location using region specific patterns [73].

Just as with other analyses in other locations, the exact nature of the environmental factors most associated with higher mosquito infection were not consistently and clearly delineated, and further studies on mosquito ecology across finely grained heterogeneous landscapes are urged to fully understand the interaction of weather and landscape in shaping mosquito population and infection dynamics. This is especially important in light of changing weather patterns associated with global climate change, as prior stability cannot be assumed. The use of local weather data at multiple locations and the integration of mosquito infection data from several sources across multiple years are important to the strength of the models presented. They allowed us to look more specifically at spatial and temporal scales that are more in keeping with the scale of virus amplification than prior studies. The ability to provide more precise spatial risk estimates is still limited, but the modeling approach presented here will help to define more clearly hypotheses that can be tested relative to the biological processes that are most closely related to the sylvatic transmission cycle.

## Competing interests

The authors declare that they have no competing interests.

## Authors' contributions

MR defined and directed the research questions and methods and was lead author in writing and revision of the manuscript. LC conceptualized and carried out the spatial model development and helped to write the manuscript. GH contributed to the background literature and helped with interpretation and conclusions. TS performed the statistical analysis for the temporal models and helped to write the results section. WB carried out data acquisition tasks, processed the spatial data and contributed to the creation of the spatial model. EW contributed to the analysis and interpretation of the data and to the conceptual design of the analysis. LH contributed to the development of the mosquito database and contributed to the interpretation of the data. TG helped with the interpretation of the data, provided revisions of the manuscript and to the development of the research questions. UK was involved with the definition of the research questions, the methods selected, and the interpretation of the data, helped to write the manuscript and provided revisions of the intellectual content.

## Supplementary Material

Additional file 1 **Details regarding the selection of covariates and patterns of mosquito infection relative to weather variables**. This file includes a graph of Mosquito infection compared to degree week, and precipitation and a correlation contour graph between degree week and mosquito infection at a range of base temperatures. Click here for file

Additional file 2 **Details regarding the temporal linear regression results**. This file has three parts: A) Scatter plots between key covariates and mosquito infection. B) Residual plots, autocorrelation function graphs, and Q-Q plots for the four linear regression models described in Table 2 in ms. C) Equations for each of the four models. Click here for file

Additional file 3 **A regression tree graphic**. An example of a regression tree graphic showing results from the 2004 model with both weather and other variables. Click here for file
