# Supplementary material for: Local impact of temperature and precipitation on West Nile virus infection in Culex species mosquitoes in northeast Illinois, USA
Source: Parasit Vectors. 2010 Mar 19;3:19. doi: 10.1186/1756-3305-3-19 (PMC2856545; doi:10.1186/1756-3305-3-19)
Supplement: Additional file 1 — Details regarding the selection of covariates and patterns of mosquito infection relative to weather variables. This file includes a graph of Mosquito infection compared to degree week, and precipitation and a correlation contour graph between degree week and mosquito infection at a range of base temperatures. [file 1756-3305-3-19-S1.PDF]

Exploratory.pdf

Title of data: Details regarding the selection of covariates and patterns of mosquito infection relative to weather variables.

This file includes A) a graph of difference in mosquito infection from 4-year average by week compared to difference in precipitation from 4-year average by week and B) a correlation contour graph between degree week and mosquito infection at a range of base temperatures from 10 to 23 C.

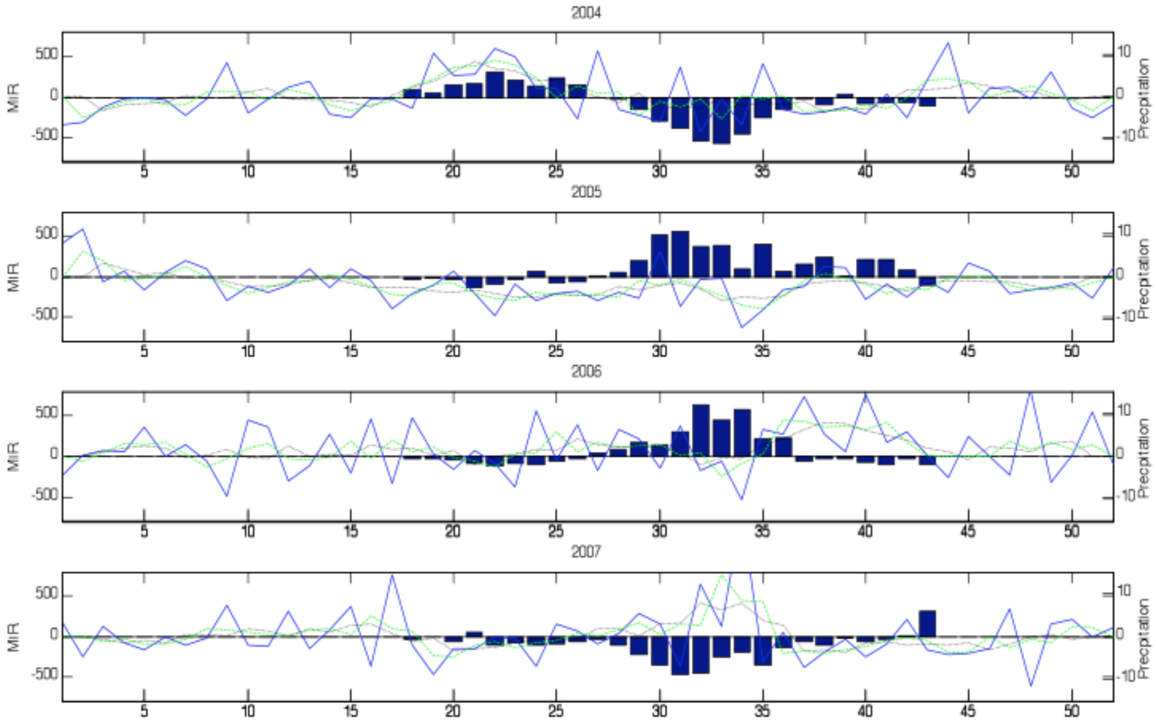

Figure A. Mosquito infection (bars) and weekly precipitation by week (lines) for the years 2004 to 2007. The values graphed are the differences from average as described in the manuscript.

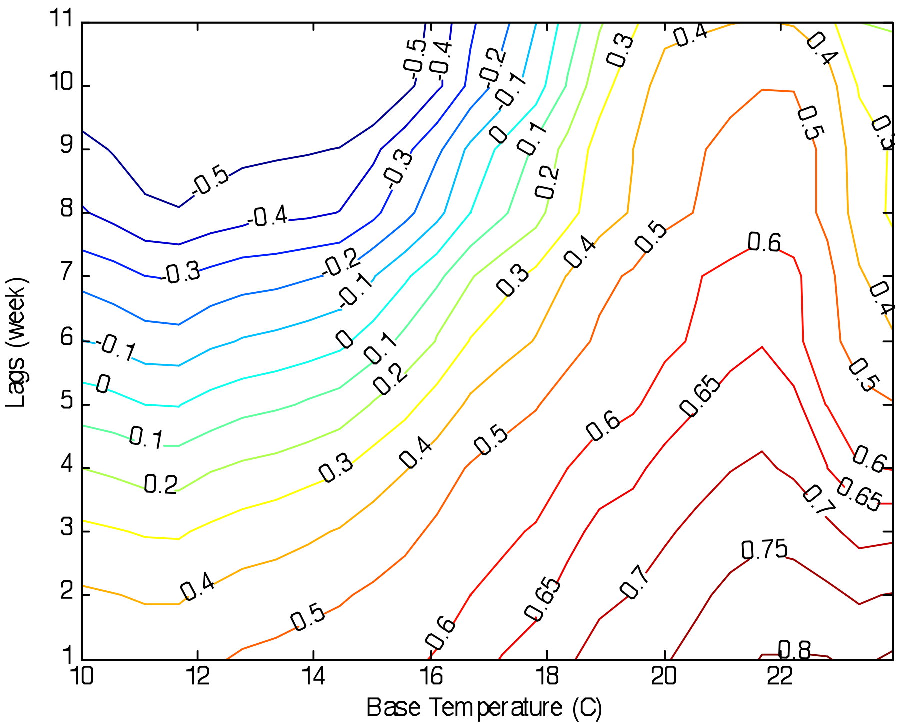

Figure B. Contour plot of correlation coefficients between the variables Degree Week and mosquito infection at multiple temporal lags and at a range of Base Temperatures between 10 and 24 degrees C.
