# Supplementary material for: Local impact of temperature and precipitation on West Nile virus infection in Culex species mosquitoes in northeast Illinois, USA
Source: Parasit Vectors. 2010 Mar 19;3:19. doi: 10.1186/1756-3305-3-19 (PMC2856545; doi:10.1186/1756-3305-3-19)
Supplement: Additional file 2 — Details regarding the temporal linear regression results. This file has three parts: A) Scatter plots between key covariates and mosquito infection. B) Residual plots, autocorrelation function graphs, and Q-Q plots for the four linear regression models described in Table 2 in ms. C) Equations for each of the four models. [file 1756-3305-3-19-S2.PDF]

Temporal.pdf

This file contains additional information about the linear regression model that modeled weekly mosquito infection rate.

A) Scatter plots between key covariates and mosquito infection

B) Residual plots, autocorrelation function graphs, and Q-Q plots for the four linear regression models described in Table 2 in ms.

C) Equations for each of the four models

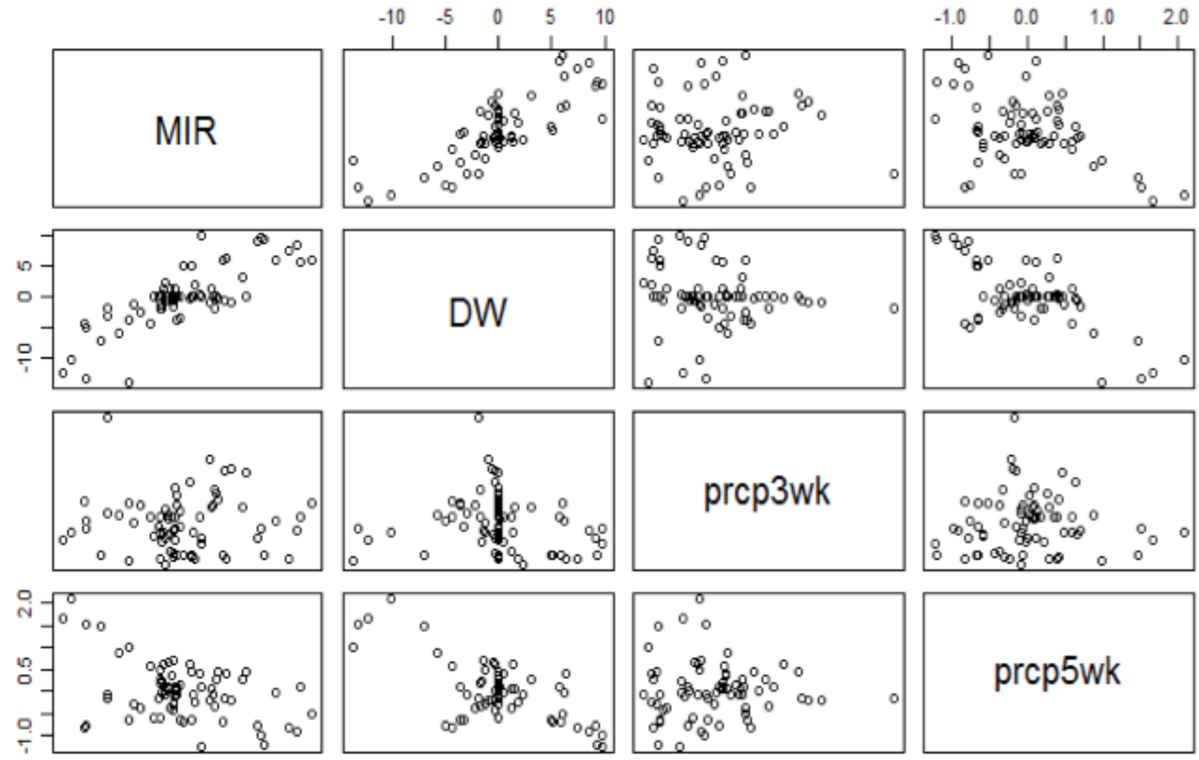

Figure A. Scatterplots between mosquito infection rate (MIR), Degree Week (DW), precipitation with 3-week average (prcp3wk), and precipitation with 5-week average (prcp5wk).

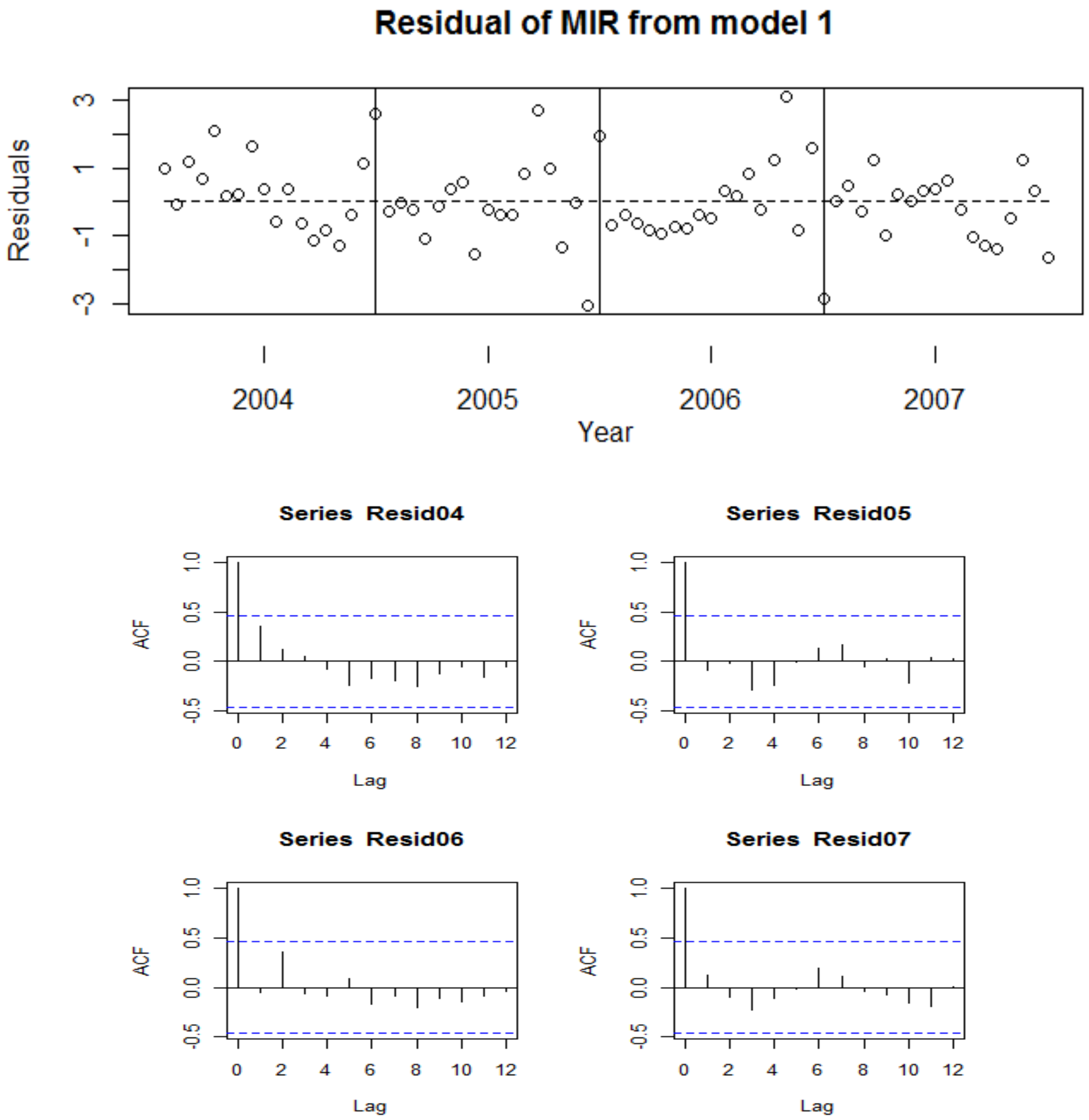

Figure B1. Residuals from model 1 (top) and the Autocorrelation Function (ACF) for the residuals for each year (bottom)

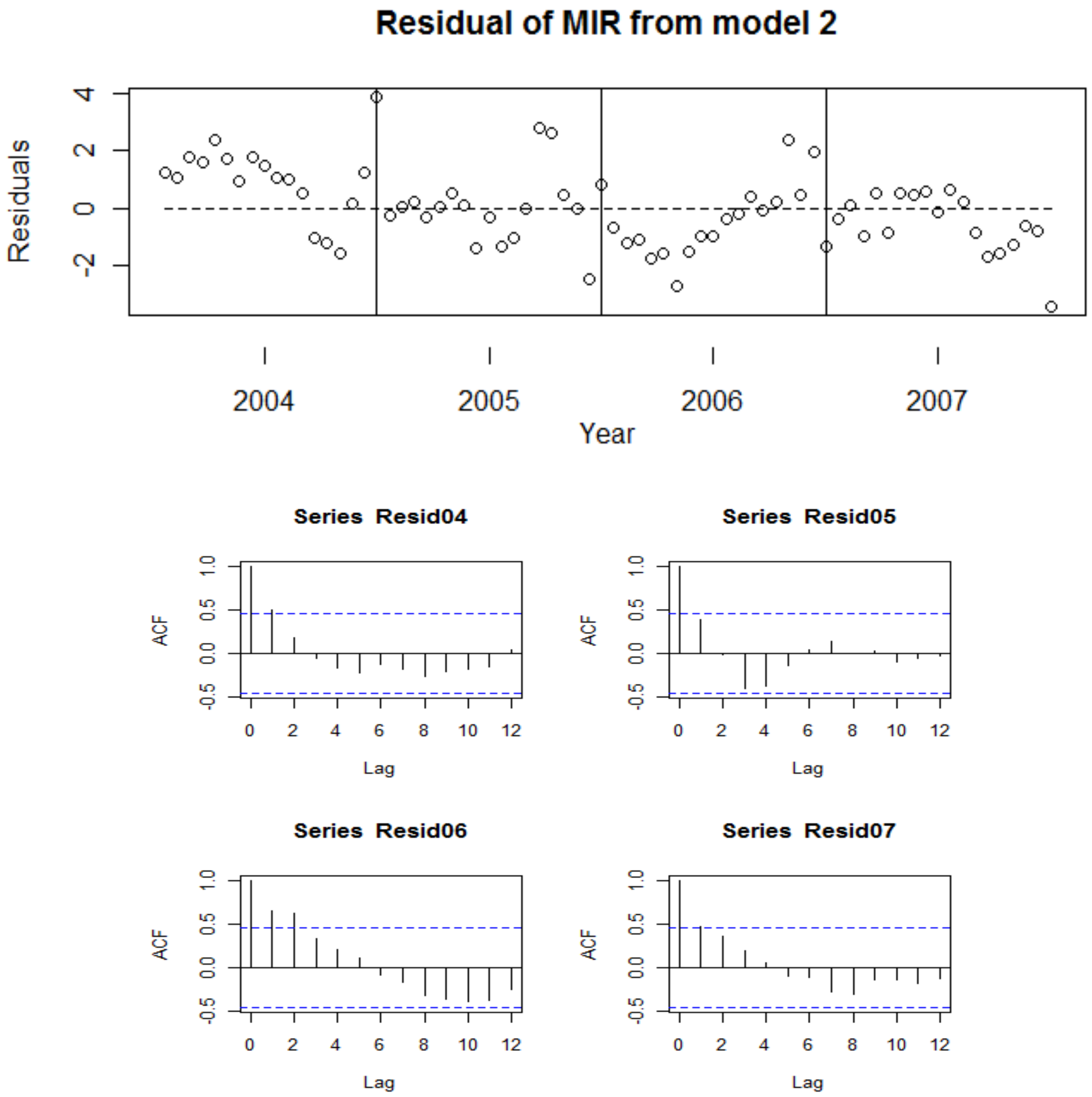

Figure B2. Residuals from model 2 (top) and the Autocorrelation Function (ACF) for the residuals for each year (bottom)

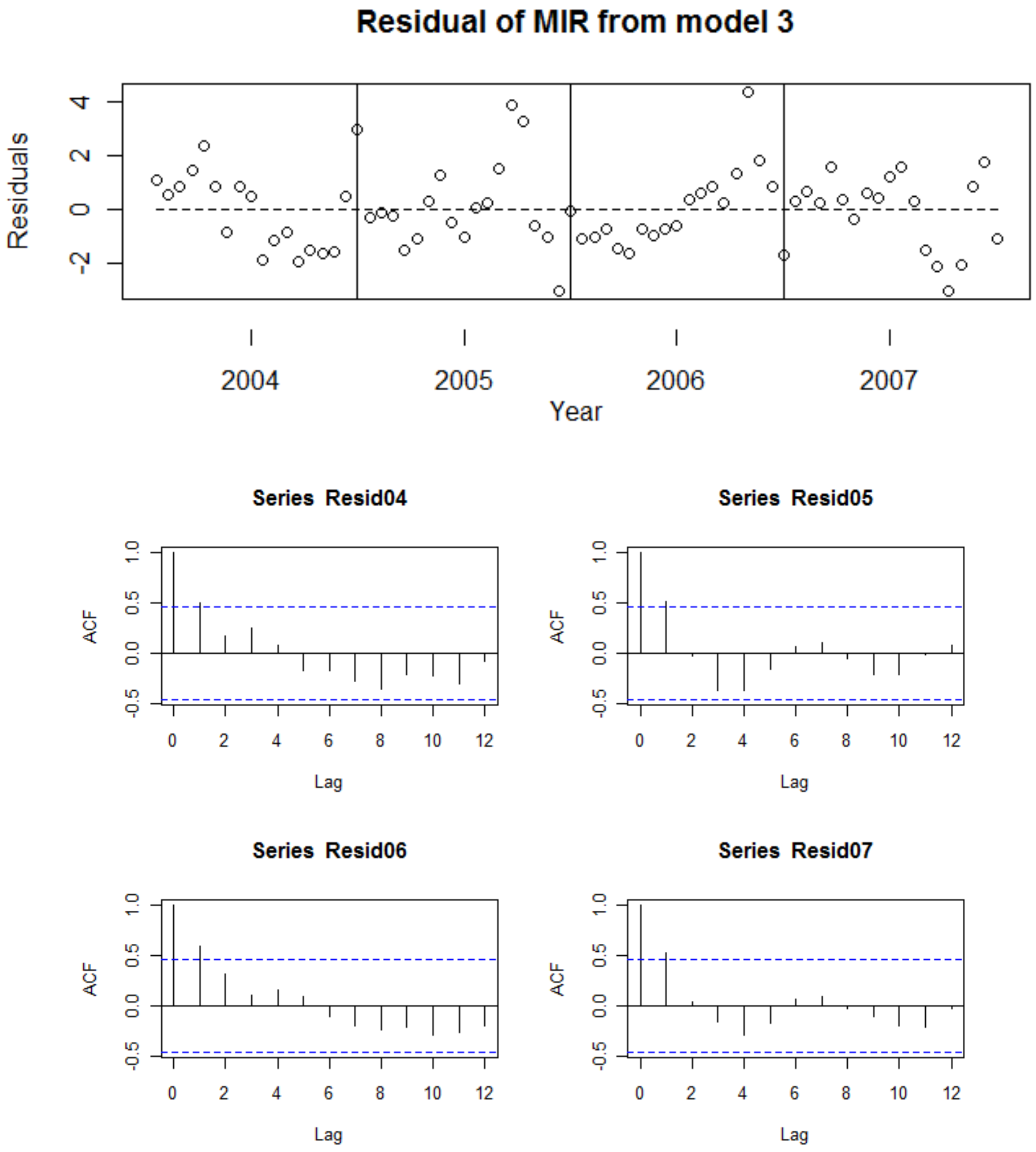

Figure B3. Residuals from model 3 (top) and the Autocorrelation Function (ACF) for the residuals for each year (bottom)

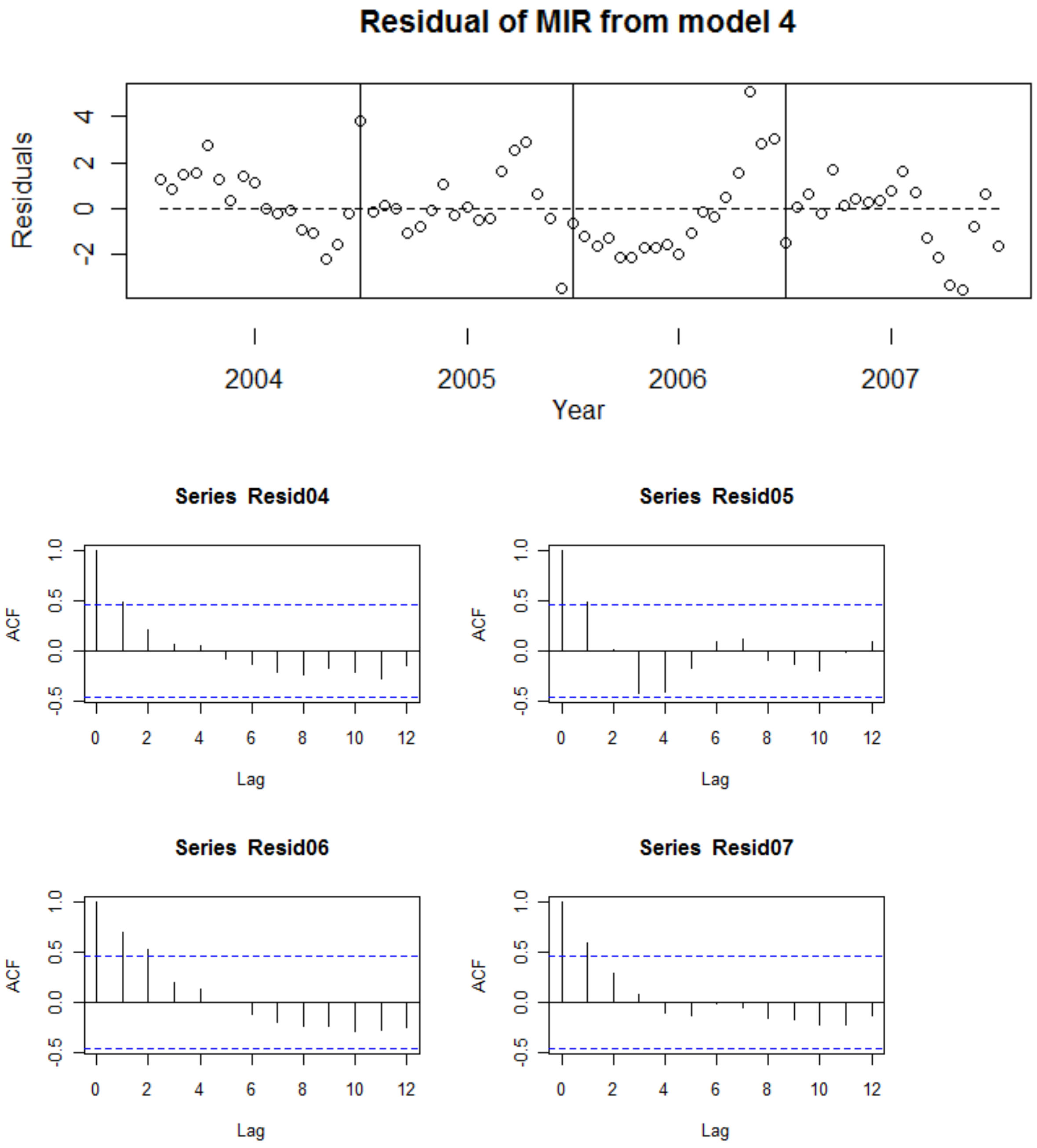

Figure B4. Residuals from model 4 (top) and the Autocorrelation Function (ACF) for the residuals for each year (bottom)

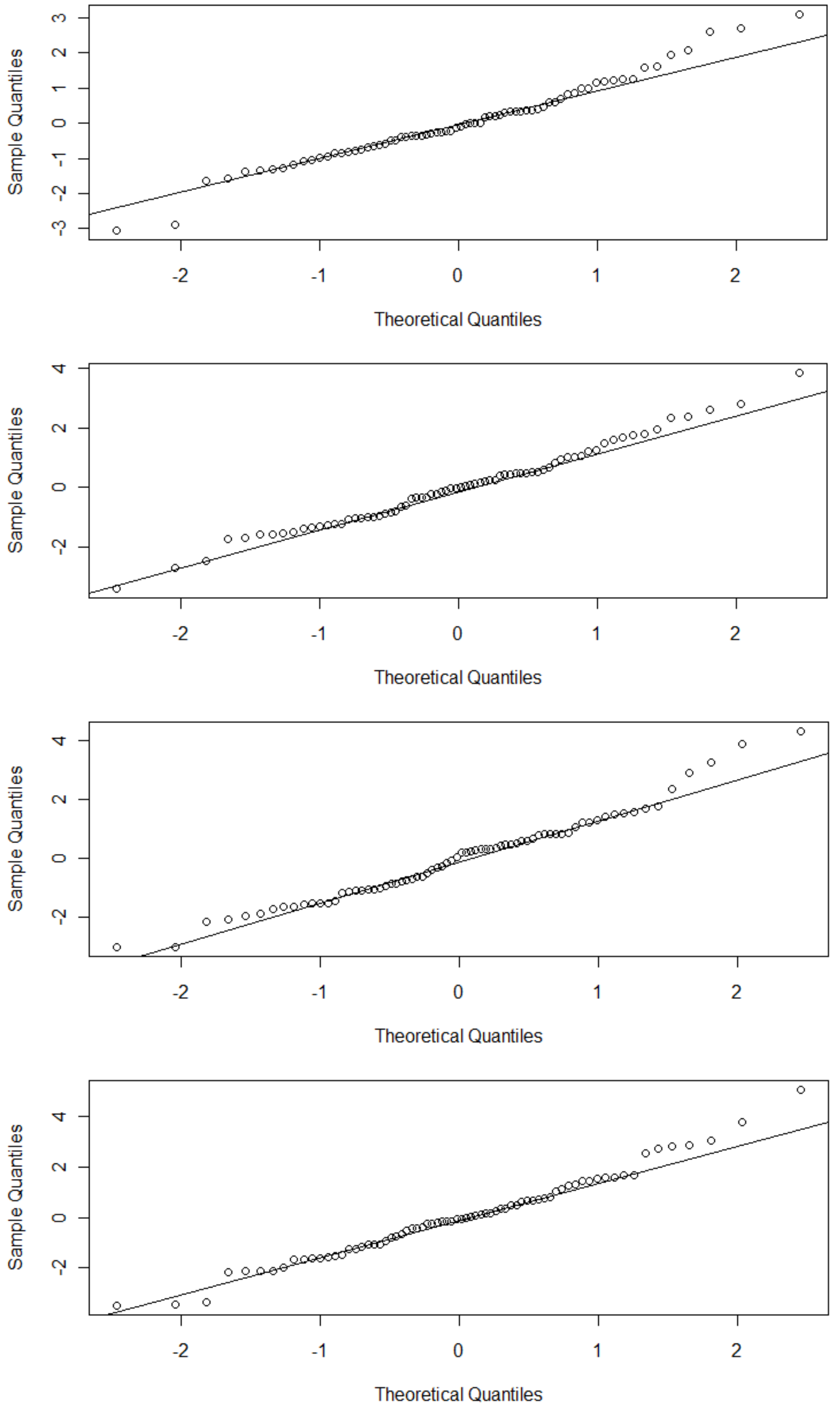

Figure B5. Q-Q plot of the residuals in model 1 to 4 (from top to bottom). All years are combined in these plots.

Model 1

$$MIR(i,j) = 8.86 * DW(i-1,j) - 19.89 * prcpann\_abn(j-1) + 0.63624 * MIR(i-1,j)$$

Model 2

$$MIR(i,j) = 23.475 * DW(i-1,j) - 40.47 * prcp\_annual(j-1) + 9.349 * prcp3wk(i-3,j)$$

Model 3

$$MIR(i,j) = 11.4 * DW(i-4,j) + 0.6 * MIR(i-2,j) - 33.4 * prcp\_annual(j-1)$$

Model 4

$$MIR(i,j) = 32.5 * DW(i-4,j) + 11.4 * prcp3wk(i-4,j) - 48.2 * prcp\_annual(j-1)$$

where the subscript i is the index for weeks, and j the index of year.

C) Equations for the four linear regression models described in the ms Table 2.
