# Supplementary material for: Local impact of temperature and precipitation on West Nile virus infection in Culex species mosquitoes in northeast Illinois, USA
Source: Parasit Vectors. 2010 Mar 19;3:19. doi: 10.1186/1756-3305-3-19 (PMC2856545; doi:10.1186/1756-3305-3-19)
Supplement: Additional file 3 — A regression tree graphic. An example of a regression tree graphic showing results from the 2004 model with both weather and other variables. [file 1756-3305-3-19-S3.PDF]

Spatial.pdf

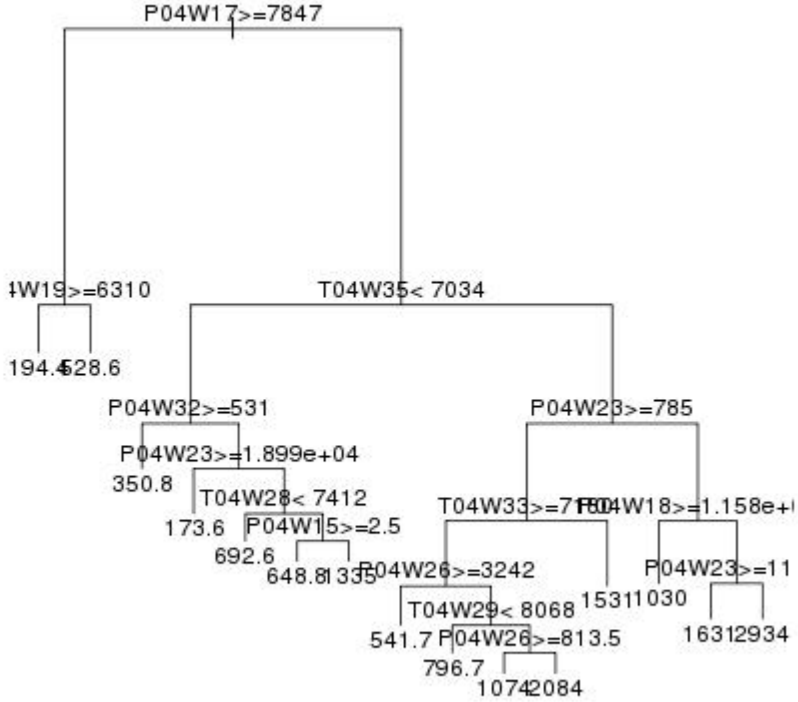


Tree graph from the Regression Tree analysis on the spatial model of mosquito infection. This model included both weather and other variables. The most important variable (the root of the tree) is precipitation at week 17, where precipitation less than or equal to about .78 inches would have higher MIR. This is followed by precipitation at week 19 and temperature at week 35. Precipitation values are measured in inches and should be divided by 1000. Temperature is measured in degrees Fahrenheit and should be divided by 100 to obtain actual values. .
